# Efficacy and safety of bedaquiline containing regimens in patients of drug-resistant tuberculosis: An updated systematic review and meta-analysis

**DOI:** 10.1016/j.jctube.2023.100405

**Published:** 2023-12-01

**Authors:** Obaid Ur Rehman, Eeshal Fatima, Abraish Ali, Umar Akram, Abdulqadir Nashwan, Faryal Yunus

**Affiliations:** a Department of Medicine, Services Institute of Medical Sciences, Lahore, Pakistan; b Department of Medicine, Dow University of Health Sciences, Karachi, Pakistan; c Department of Medicine, Allama Iqbal Medical College, Lahore, Pakistan; d Hamad Medical Corporation, Doha, Qatar; e Department of Pathology, Services Institute of Medical Sciences, Lahore, Pakistan

**Keywords:** Bedaquiline, Efficacy, Drug resistance, Meta-analysis, Tuberculosis, Multidrug resistant

## Abstract

**Background:**

Tuberculosis is an infectious disease caused by *Mycobacterium tuberculosis* and leads to serious complications if left untreated. Some strains of *Mycobacterium tuberculosis* are multi-drug resistant and require treatment with newer drugs. Bedaquiline based treatment regimens have been used in patients who are diagnosed with drug resistant tuberculosis. The aim of this study is to assess the efficacy and safety profile of bedaquiline-based treatment regimens using a systematic review of existing literature and *meta*-analysis.

**Methods:**

In this study, an electronic search was carried out on PubMed, ScienceDirect, and Cochrane library to find relevant literature from March 2021 onwards. Random-effects model was used to assess pooled treatment success rate and 95 % CIs. p-value of <0.05 was suggestive of publication bias. The review is registered with PROSPERO: CRD42023432748.

**Results:**

A total of 543 articles were retrieved by database searching, out of which 12 new studies met the inclusion criteria. The total number of articles included in the review was 41 including 36 observational studies (having a total of 9,934 patients) and 5 experimental studies (having a total of 468 patients). The pooled treatment success rate was 76.9 % (95 % CI, 72.9–80.4) in the observational studies and 81.7 % (95 % CI, 67.2–90.7) in the experimental studies. Further subgroup analysis was done on the basis of treatment regimens containing bedaquiline only and treatment regimens containing bedaquiline and delamanid. The pooled treatment success rate in the studies consisting of patients who were treated with regimens containing bedaquiline only was 78.4 % (95 % CI, 74.2–82.1) and 73.6 % (95 % CI, 64.6–81.0) in studies consisting of patients who were treated with regimens containing bedaquiline and delamanid. There was no evidence of publication bias.

**Conclusions:**

In patients of drug resistant tuberculosis having highly resistant strains of *Mycobacterium tuberculosis* undergoing treatment with bedaquiline-based regimen demonstrate high rates of culture conversion and treatment success. Moreover, the safety profile of bedaquiline-based regimens is well-established in all studies.

## 1 Introduction

It is believed that about one-fourth of the world's population has been exposed to tuberculosis bacteria. Globally, tuberculosis (TB) ranks as the thirteenth most prevalent cause of mortality, and it stands as the second most significant infectious agent (after COVID-19) responsible for fatalities, surpassing both HIV and AIDS. In 2021, the global mortality rate due to tuberculosis (TB) reached 1.6 million individuals, while approximately 10.6 million individuals were afflicted with the disease [1]. In recent years, the emergence of drug resistance in tuberculosis (TB) has been attributed to various factors, including the inappropriate utilization of TB medications, such as their incorrect and inadequate prescription by healthcare professionals, the availability of substandard drugs, and non-compliance of patients leading to discontinuation of therapy [1]. Multidrug-resistant tuberculosis (MDR-TB) is a variant of tuberculosis (TB) that is characterized by bacterial strains exhibiting resistance to isoniazid and rifampicin, which are recognised as the two most efficacious first-line drugs for the treatment of TB [2]. MDR-TB which is also resistant to any fluoroquinolone was defined as Pre-Extensively Drug-Resistant Tuberculosis (Pre-XDR-TB) while MDR-TB which is also resistant to any fluoroquinolone and at least one of the injectables second line drugs was defined as Extensively Drug-Resistant Tuberculosis (XDR-TB) [3].

Multidrug-resistant tuberculosis (MDR-TB) poses a significant challenge to public health and represents a health security concern, necessitating intricate and financially burdensome treatment protocols [1]. On a global scale, the number of incident cases of multidrug-resistant tuberculosis (MDR-TB) in 2021 was approximately 450,000, reflecting a 3.1 % increase compared to the 437,000 cases reported in 2020 [2]. The treatment outcomes for MDR-TB are suboptimal, as evidenced by a success rate of approximately 50 % among patients undergoing treatment [4]. XDR-TB poses significant challenges in terms of treatment, necessitating longer and more complex therapeutic regimens compared to MDR-TB. The available treatment options are characterized by limitations in terms of efficacy, high costs, and potential toxicity [5]. In 2018, the World Health Organisation (WHO) reported a global treatment success rate of 39 % XDR-TB [6].

Bedaquiline, a recently developed drug, has demonstrated notable efficacy in the management of MDR-TB. The drug in question represents a novel therapeutic agent that operates through a distinct mechanism of action in comparison to the currently available anti-tuberculosis medications. The efficacy of bedaquiline has been demonstrated in reducing the duration of treatment for MDR-TB and enhancing the rate of treatment success [7], [8], [9]. Bedaquiline was included in the World Health Organization's recommended all-oral regimen as a substitute for injectable treatments in patients with MDR-TB [7].

Several observational [10], [11], [12], [13]. and experimental studies [14], [15], [16], [17]. have been carried out to study the efficacy of bedaquiline against MDR-TB. A *meta*-analysis was conducted in 2021 that pooled the results of these studies [21]. However, results from additional well-designed observational and experimental studies were published [19], [20], [40], [41], [42], [43], [44], [45], [46], [47], [48], [49], [50], [51], [52], [53], [54], [55], [56], [57], [58], [59], [60], [61], [62], [63], [64], [65], [66], [67], [68], [69]. To better analyze the effectiveness and safety of bedaquiline in MDR-TB, we conducted an updated *meta*-analysis.

## 2 Methods

### 2.1 Search Strategy

This *meta*-analysis was conducted and reported in conformity with the Cochrane and PRISMA (Preferred Reporting Items for Systematic Review and Meta‐Analyses) guidelines [22]. We searched for original studies reporting the efficacy of dosing regimens consisting of bedaquiline in patients of MDR-TB/ pre-XDR TB/ XDR TB confirmed after culture or drug-susceptibility testing. An electronic search was conducted on Cochrane CENTRAL, MEDLINE, Scopus, and ClinicalTrials.gov databases from March 2021 till August 2023. Detailed search strings for each database are presented in the Supplementary Table S1. No restriction regarding country, race or publication language were set. Reference list from related main studies and review articles were also checked for additional relevant studies. The review is registered with PROSPERO: CRD42023432748.

### 2.2 Study selection and eligibility Criteria

Articles were selected if they met the following prespecified eligibility criteria: (i) single arm observational or experimental studies (ii) patients who have been diagnosed with MDR-TB per WHO criteria [23]; (iii) patients who have been treated with dosing regimens containing bedaquiline; and (iv) treatment success (i.e, cultural conversion) was reported. The exclusion criteria included (i) non human studies, case series, editorials, abstracts, reviews, comments and letters, expert opinions, studies without original data, duplicate publications, and articles describing tuberculosis patients who were recruited without a confirmed bacteriological diagnosis.

TB caused by *Mycobacterium tuberculosis* strains and fulfilling the definition of MDR-TB or rifampin-resistant TB and also resistant to any fluoroquinolone was defined as Pre-Extensively Drug-Resistant Tuberculosis Pre-XDR-TB. Tuberculosis caused by *M. tuberculosis* strains and fulling the definition of MDR-TB or rifampin-resistant TB and also resistant to any fluoroquinolone and at least one additional group A drug was defined as Extensively Drug-Resistant Tuberculosis (XDR-TB) [3].

Treatment outcomes were recorded in accordance with adapted definitions of those given in the WHO guidelines, as follows: treatment success, defined as the combination of the number of patients who completely recovered and that of those who completed their treatment; death, defined as mortality from any cause while on treatment; and treatment failure defined as unsuccessful treatment, as determined by positive cultures at the end of the treatment regimen [24].

### 2.3 Data extraction and Quality Assessment

The articles retrieved from the systematic search of databases were exported to EndNote Reference Library Software, and duplicates were removed. Two independent reviewers (Nashwan.A. and Ali.A.) first screened the remaining articles on the basis of title and abstract, after which the full text was reviewed to assess relevance. Any discrepancies were addressed through discussion until a consensus was reached. The primary outcome of interest was the treatment success rate i.e., the number of patients who completed their treatment and also achieved a cure according to WHO guidelines. The following data were extracted from all eligible studies: first author’s name; year of publication; study duration; type of study; country or countries where the study was conducted; the number of patients with MDR-TB; patient age; treatment protocols (treatment regimens and duration of treatment); demographics; adverse effects; drug resistance status; and outcomes. Quality assessment of the included studies was done using two different assessment tools (checklists): one for observational and one for experimental studies adopted from the Joanna Briggs Institute. Critical Appraisal Tools [26]. Items such as study population, measure of exposures, confounding factors, extent of outcomes, follow-up data, and statistical analysis were evaluated.

### 2.4 Data Analysis

Comprehensive Meta-Analysis software, version 3.3 (Biostat Inc., Englewood, NJ, USA) was used to perform statistical analyses. The pooled success rate with 95 % CI was assessed using the random-effects model. The random-effects model was used because of the estimated heterogeneity of the true effect sizes. We conducted a separate analysis for observational and experimental studies respectively, Additionally, subgroup analyses stratified by type of study and treatment regimen [bedaquiline-based (BDQ) regimen, bedaquiline and delamanid-based (BDQ + DLM) regimen] were performed to minimize heterogeneity. Heterogeneity across studies was evaluated using Higgins I^2^ where a value of 50 % and more was considered. Publication bias was statistically assessed by using Begg’s test, as well as funnel plots, a value of p < 0.05 being considered indicative of statistically significant publication bias and funnel plot asymmetry being suggestive of bias [25].

## 3 Results

**Fig. 1:**
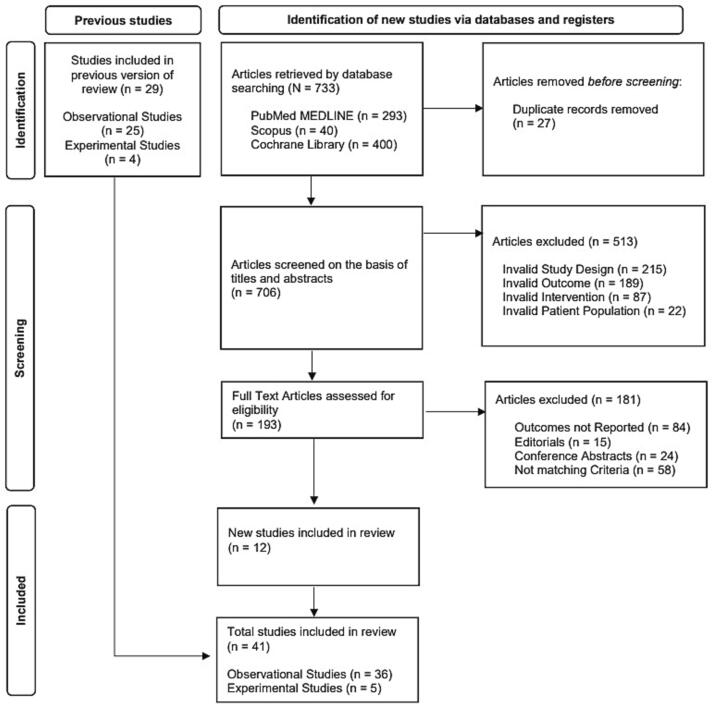
PRISMA Flow Diagram showing search strategy adopted for conducting this review.

We searched the databases for articles after March 2021. As shown in Fig. 1*,* the electronic search across three databases yielded a total of 733 articles. After the removal of duplicates, the titles and abstracts of 706 articles were screened. Of those, 193 were selected for a full-text review. After a full-text review, 12 new studies were chosen for updating the previous version of the systematic review and *meta*-analysis. In total, 41 studies are included in the updated version of this review. The studies [10], [11], [12], [13], [14], [15], [16], [17], [18], [19], [20], [40], [41], [42], [43], [44], [45], [46], [47], [48], [49], [50], [51], [52], [53], [54], [55], [56], [57], [58], [59], [60], [61], [62], [63], [64], [65], [66], [67], [68], [69] were divided into two groups: 36 observational studies including a total of 9,934 patients, and 5 experimental studies including a total of 468 patients. The patient’s mean age was 36.2 years. The treatment duration ranged from a minimum of 5 months to a maximum of 22 months with most studies having a treatment duration of 6 months. Out of the patients, 3510 were HIV+, and 6,999 patients had prior TB treatment. The earliest study was published in 2014, and the latest study was published in 2023. Table 1 summarizes the baseline characteristics of patients included in individual studies.

**Table 1:** Studies Included in the Meta Analysis.

**Author**	**Year**	**Country**	**Type of study**	**Mean/ median age**	**HIV+, n (%)**	**Previously treated for TB**	**TB disease**	**No. of patients receiving BDQ**	**Other drugs included in the regimen**	**Duration of treatment (months)**	**Outcomes**		
											**Treatment success**	**Treatment failure**	**Death**
Koirala et al.	2021	Multicenter	PC	39	27 (5.7)	329	MDR/XDR	383	WHO-recommended regimen*	6	284	11	25
Kwon et al.	2021	South Korea	RC	49	0	19	Pre-XDR/ XDR	28	DLM, LZD, CFZ, MEM/CLV, CYC	6	23	2	1
Shi et al.	2021	China	RC	49.8	N/R	186	MDR	72	FLQs, LZD, CFZ, CYC	6	197	4	0
							pre-XDR	78					
							XDR	64					
Gao et al	2021	China	RC	40	1 (0.6)	168	MDR	39	FLQs, LZD, CFZ, CYC	6	151	23	3
							pre-XDR	56					
							XDR	82					
Barvaliya et al	2020	India	PC	32	N/R	110	pre-XDR	87	FLQs, LZD, CFZ	5.5	102	10	14
							XDR	40					
Kashongwe et al.	2020	Congo	RC	32.4	3 (9.4)	23	pre-XDR	29	FLQs, LZD, CFZ, CYC	20	17	0	15
							XDR	3					
Das et al.	2020	India	RC	Children	0	N/R	Pre-XDR/ XDR	13	DLM, LZD, CFZ	22	12 or 13	N/R	N/R
Lee et al.	2020	South Korea	RC	49.8	1 (1.4)	49	MDR	13	DLM, FLQs, LZD, CFZ, CYC	5.5	42	1	4
							pre-XDR	41					
							XDR	20					
Kimet al.	2020	South Korea	RC	33	9 (3.5)	254	MDR	159	AMGs, FLQs, LZD, CYC	6	139/225	35/225	15/225
							pre-XDR	51					
							XDR	44					
Mase et al	2020	USA	RC	43.5	1 (7)	5	MDR	7	WHO-recommended regimen*	5.5	12	N/R	1
							pre-XDR	4					
							XDR	3					
Olayanju et al.	2020	South Africa	PC	33	42 (51)	40	MDR	5	AMGs, FLQs, LZD, CFZ, TRD	6	52	N/R	N/R
							pre-XDR	10					
							XDR	67					
				34	22 (55)	29	MDR	6	DLM, AMGs, FLQs, LZD, CFZ, TRD	6	27	N/R	N/R
							pre-XDR	15					
							XDR	19					
Salhotra et al.	2020	India	PC	Range: 18–50	8 (1.3)	600	MDR	524	AMGs, FLQs, CFZ	6	513	N/R	73
							XDR	96					
Chesov et al.	2020	Moldova	RC	37	17 (14.9)	58	MDR	114	AMGs, FLQs, CYC, PZA	6	63	31	10
Kang et al.	2020	South Korea	RC	51.7	0	55	MDR	43	AMGs, FLQs, LZD, CYC	6	86	1	13
							pre-XDR	47					
							XDR	17					
				47.7	1 (1.5)	47	MDR	8	DLM, AMGs, FLQs, LZD, CYC	6	58	3	3
							pre-XDR	37					
							XDR	22					
Sarin et al.	2019	India	PC	Range: 21–33	0	N/R	MDR/ Pre-XDR/ XDR	42	DLM, FLQs, LZD, CFZ, IMP	6	25	N/R	10
Kempker et al.	2019	USA	PC	37.3	2 (3)	8	MDR/XDR	64	FLQs, LZD, CFZ, CYC, IMP	5.5	42	1	0
Taune et al.	2019	New Guinea	RC	39	1 (1.3)	33	MDR	55	AMGs, FLQs, LZD, CFZ, CYC, PZA	6	72	N/R	5
							pre-XDR	10					
							XDR	12					
Ferlazzo et al	2018	Armenia, India, South Africa	RC	32.5	11 (39)	4	MDR	2	DLM, FLQs, LZD, CFZ, IMP	6	22	N/R	1
							pre-XDR	12					
							XDR	14					
Hewison et al.	2018	Armenia, Georgia	RC	40.5	4 (4.8)	N/R	MDR	6	FLQs, LZD, CFZ, IMP	6	48	6	10
							pre-XDR	36					
							XDR	40					
Ndjeka et al.	2018	South Africa	PC	34	134 (67)	N/R	pre-XDR	122	FLQs, LZD, CFZ	6	146	9	25
							XDR	78					
Zhao et al.	2018	South Africa	PC	Range: 35–49	110 (68)	N/R	MDR	162	FLQs, PZA, ETH, hINH, ETM, TRD	6	111/146	7/119	11/145
Kim et al	2018	South Korea	RC	52	N/R	N/R	MDR/ Pre-XDR/ XDR	39	FLQs, LZD, CFZ	5.6	24	N/R	N/R
Achar et al	2017	South Africa, Tajikistan, Uzbekistan, Belarus	PC	Children	0	N/R	Pre-XDR/ XDR	23	FLQs, LZD, CFZ, IMP	6	23	0	0
Guglielmetti et al.	2017	France	RC	38	2 (4.4)	34	MDR/ Pre-XDR/ XDR	45	AMGs, FLQs, LZD, CFZ, CYC, PZA, ETH, ETM	6	36	1	3
Borisov et al.	2017	Multicenter	RC	35	94 (22.1)	334	MDR	233	AMGs, FLQs, LZD, CFZ, IMP	5.5	176/247	18/247	33/247
							XDR	195					
Conradie et al.	2020	South Africa	CT	35	56 (51)	N/R	MDR	38	LZD, PMD	6	98	2	7
							XDR	71					
Tweed et al.	2019	South Africa, Tanzania, Uganda	CT	34	25 (42)	N/R	RR	60	FLQs, PZA, PMD	6	58	N/R	0
Pym et al.	2016	Multicenter	CT	32	8 (4)	177	MDR	124	AMGs, FLQs, CYC, PZA, ETH	6	163	N/R	N/R
							pre-XDR	44					
							XDR	37					
Diacon et al.	2014	Multicenter	CT	32	5 (8)	N/R	MDR	66	AMGs, FLQs, CYC, PZA, ETH	6	52	N/R	N/R
Moodlier et al.	2021	Multicenter	CT	16, 7	N/R	29	MDR	21	WHO Recommended Regimen*	6	13/28	2/28	N/R
							RR	9					
Trevisi et al.	2023	Multicenter	RC	34	95 (6.5)	1181	RR/MDR	1468	FLQs, LZD, CFZ, CYC	6–24	1288	N/R	N/R
Shim et al.	2023	South Korea	PC	18–65	N/R	57	MDR	60	WHO Recommended Regimen*	5.4	49/87	0/87	13/87
							pre-XDR	5					
							XDR	23					
Pai et al.	2022	South Africa	RC	12–65	2754	2328	MDR	1526	WHO Recommended Regimen*	6	2501/3799	108/3739	577/3739
							pre-XDR	1039					
							XDR	1182					
Nguyen et al.	2023	Vietnam	PC	41	1	40	RR- TB	106	AMGs, FLQs, FLQs, CYC, LZD, PZA, Prothionamide, ETM, hINH	9–11	95	4	1
Nguyen et al.(2)	2022	Vietnam	PC	43.7	3	86	Pre-XDR TB	99	FLQs, LZD, CFZ, PZA	6–9	75	2	7
Zhang et al.	2022	East China		37	N/R	93	RR-TB	3	PZA, LZD, CFZ, CYC, Protionamide	6	94	3	1
							MDR	74					
							XDR	25					
Padmapriyadarsini et al.	2023	India	PC	27	N/R	152	Pre-XDR TB	152	FLQs, PZA, Isoniazid, ETM, SLI	6	139	N/R	4
Huegra et al.	2022	Multicenter	PC	36	73	435	Pre -XDR TB	381	LZD, CFZ, PZA, Carbapenem, FLQs, CYC, ETM	6	358	33	41
							MDR-TB/RR-TB	52					
							N/A	39					
Wu et al.	2022	China	RC	15.6	N/R	1	RR	4	LZD, CYC, PZA, FLQs, ETM, Isoniazid, Prothionamide, CFZ	6	10	N/R	N/R
							MDR	4					
							XDR	2					
Li et al.	2021	China	RC	31	0	35	MDR	14	LZD, PZA, Prothionamide, CFZ, Amikacin, FLQs, ETM, CYC	6	21	2	1
							pre-XDR	16					
							XDR	5					
Hwang et al.	2021	South Korea	RC	51	0	N/R	RR/MDR	44	FLQs, LZD	5.7	90	2	18
							Pre XDR	55					
							XDR	20					

PC: prospective cohort; RC: retrospective cohort; CT: clinical trial; BDQ: bedaquiline; DLM: delamanid; FLQs: fluoroquinolones; LZD: linezolid; CFZ: clofazimine; CYC: cycloserine; AMGs: aminoglycosides; MEM/CLV: meropenem-clavulanate; TRD: terizidone; IMP: imipenem; ETH: ethionamide; hINH: high-dose isoniazid; ETM: ethambutol; PZA: pyrazinamide; PMD: pretomanid; SLI: Second Line Injectable; MDR: multidrug-resistant; XDR: extensively drug-resistant; RR: rifampin-resistant; and N/R: not reported.

* WHO Recommended Regimen: 6-Month Bedaquiline, Pretiomanid, Linezolid, and Moxifloxacin (BPaLM).

### 3.1 Quality of the included Studies

The checklist for observational studies [26] showed that the observational studies included had a low risk of bias (Table 2). On the other hand, the included experimental studies had a high risk of bias for participant assignment, randomization, group concealment, and assessor blinding (Table 3) according to the checklist for experimental studies [26].

**Table 2:** Quality assessment of the observational studies included in the *meta*-analysis.

Authors	1	2	3	4	5	6	7	8	9	10	11
Koirala et al. (2021)	N/A	N/A	Yes	No	No	Yes	Yes	Yes	Yes	Yes	Yes
Kwon et al. (2021)	N/A	N/A	Yes	No	No	Yes	Yes	Yes	Yes	Yes	Yes
Shi et al. (2020)	N/A	N/A	Yes	No	No	Yes	Yes	Yes	Yes	Yes	Yes
Gao et al. (2020)	N/A	N/A	Yes	No	No	Yes	Yes	Yes	Yes	Yes	Yes
Barvaliya et al. (2020)	N/A	N/A	Yes	No	No	Yes	Yes	Yes	Yes	Yes	Yes
Kashongwe et al. (2020)	N/A	N/A	Yes	No	No	Yes	Yes	Yes	Yes	Yes	Yes
Das et al. (2020)	N/A	N/A	Yes	No	No	Yes	Yes	Yes	Yes	Yes	Yes
Lee et al. (2020)	N/A	N/A	Yes	No	No	Yes	Yes	Yes	Yes	Yes	Yes
Mase et al. (2020)	N/A	N/A	Yes	No	No	Yes	Yes	Yes	Yes	Yes	Yes
Kim et al. (2020)	N/A	N/A	Yes	No	No	Yes	Yes	Yes	Yes	Yes	Yes
Olayanju et al. (2020)	N/A	N/A	Yes	No	No	Yes	Yes	Yes	Yes	Yes	Yes
Salhotra et al. (2020)	N/A	N/A	Yes	No	No	Yes	Yes	Yes	Yes	Yes	Yes
Cheesov et al. (2021)	Yes	Yes	Yes	No	No	Yes	Yes	Yes	Yes	Yes	Yes
Kang et al. (2020)	Yes	Yes	Yes	No	No	Yes	Yes	Yes	Yes	Yes	Yes
Sarin et al. (2019)	N/A	N/A	Yes	No	No	Yes	Yes	Yes	Yes	Yes	Yes
Kempker et al. (2019)	Yes	Yes	Yes	No	No	Yes	Yes	Yes	Yes	Yes	Yes
Taune et al. (2019)	Yes	Yes	Yes	No	No	Yes	Yes	Yes	Yes	Yes	Yes
Ferlazzo et al. (2018)	N/A	N/A	Yes	No	No	Yes	Yes	Yes	Yes	Yes	Yes
Hewsion et al. (2018)	N/A	N/A	Yes	No	No	Yes	Yes	Yes	Yes	Yes	Yes
Ndjeka et al. (2018)	N/A	N/A	Yes	No	No	Yes	Yes	Yes	Yes	Yes	Yes
Zhao et al. (2018)	Yes	Yes	Yes	No	No	Yes	Yes	Yes	Yes	Yes	Yes
Kim et al. (2018)	Yes	Yes	Yes	No	No	Yes	Yes	Yes	Yes	Yes	Yes
Achar et al. (2017)	N/A	N/A	Yes	No	No	Yes	Yes	Yes	Yes	Yes	Yes
Guglielmetti et al. (2017)	N/A	N/A	Yes	No	No	Yes	Yes	Yes	Yes	Yes	Yes
Borisov et al. (2017)	N/A	N/A	Yes	No	No	Yes	Yes	Yes	Yes	Yes	Yes
Trevisi et al. (2023)	N/A	N/A	Yes	No	No	Yes	Yes	Yes	Yes	Yes	Yes
Shim et al.	Yes	Yes	Yes	No	No	Yes	Yes	Yes	Yes	Yes	Yes
Pai et al.	Yes	Yes	Yes	No	No	Yes	Yes	Yes	Yes	Yes	Yes
Nguyen et al.	N/A	N/A	Yes	No	No	Yes	Yes	Yes	Yes	Yes	Yes
Nguyen et al. (2)	N/A	N/A	Yes	No	No	Yes	Yes	Yes	Yes	Yes	Yes
Zhang et al.	Yes	Yes	Yes	No	No	Yes	Yes	Yes	Yes	Yes	Yes
Padmapriyadarsini et al.	N/A	N/A	Yes	No	No	Yes	Yes	Yes	Yes	Yes	Yes
Huerga et al.	N/A	N/A	Yes	No	No	Yes	Yes	Yes	Yes	Yes	Yes
Wu et al.	N/A	N/A	Yes	No	No	Yes	Yes	Yes	Yes	Yes	Yes
Li et al.	N/A	N/A	Yes	No	No	Yes	Yes	Yes	Yes	Yes	Yes
Hwang et al.	Yes	Yes	Yes	No	No	Yes	Yes	Yes	Yes	Yes	Yes

**Questions:**

1. Were the two groups similar and recruited from the same population?

2. Were the exposures measured similarly to assign people to both exposed and unexposed groups?

3. Was the exposure measured in a valid and reliable way?

4. Were confounding factors identified?

5. Were strategies to deal with confounding factors stated?

6. Were the groups/participants free of the outcome at the start of the study?

7. Were the outcomes measured in a valid and reliable way?

8. Was the follow-up time reported and long enough for outcomes to occur?

9. Was follow-up complete, and, if not, were the reasons for loss to follow-up described and explored?

10. Were strategies to address incomplete follow-up utilized?

11. Was appropriate statistical analysis used?

**Table 3:** Quality assessment of the experimental studies included in the *meta*-analysis.

**Author**	**1**	**2**	**3**	**4**	**5**	**6**	**7**	**8**	**9**	**10**	**11**	**12**	**13**
Conradie et al.	No	N/A	N/A	No	No	No	No	Yes	Yes	N/A	Yes	Yes	No
Tweed et al.	Yes	No	Yes	No	No	No	No	Yes	Yes	Yes	Yes	Yes	Yes
Pym et al.	No	No	N/A	No	No	No	No	Yes	Yes	N/A	Yes	Yes	No
Diacon et al.	Yes	Yes	Yes	Yes	Yes	Yes	No	Yes	Yes	Yes	Yes	Yes	Yes
Moodlier et al.	No	No	Yes	No	No	No	No	Yes	Yes	Yes	Yes	Yes	Yes

**Questions:**

1. Was true randomization used for assignment of participants to treatment groups?

2. Was allocation to treatment groups concealed?

3. Were treatment groups similar at baseline?

4. Were participants blind to treatment assignment?

5. Were those delivering treatment blind to treatment assignment?

6. Were outcome assessors blind to treatment assignment?

7. Were treatment groups treated identically other than the intervention of interest?

8. Was follow-up complete, and, if not, were differences between groups in terms of their follow-up adequately described and analyzed?

9. Were participants analyzed in the groups to which they were randomized?

10. Were outcomes measured in the same way for treatment groups?

11. Were outcomes measured in a reliable way?

12. Was appropriate statistical analysis used?

13. Was the trial design appropriate and were any deviations from the standard randomized controlled trial design accounted for in the conduct and analysis of the trial?

### 3.2 Outcomes in the observational Studies

**Fig. 2:**
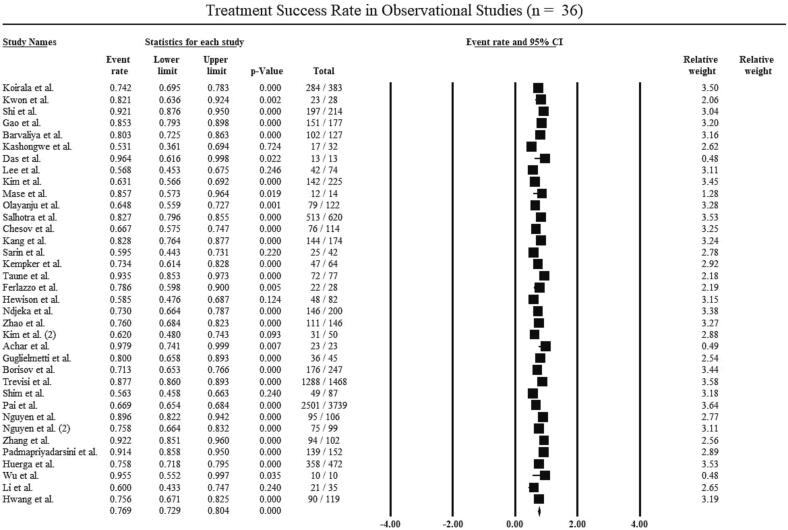
Treatment success rate of the pooled observational studies.

The treatment success rate of the pooled observational studies (n = 36) included in this review was significant 76.9 % [(95 % CI, 72.9–80.4); p = 0.00; I^2^ = 92 %; Fig. 2]. The Begg’s Test showed that there was no publication bias (p-value > 0.05; Table 5).

**Table 4:** Adverse Events (AEs) in the studies included in the *meta*-analysis.

**Author**	**QTc prolongation**	**Liver disease/ Elevated liver enzyme**	**Renal failure/ Increased creatinine levels**	**Optic neuropathy/ Blurred vision**	**Ototoxicity/ Hearing loss**	**Hematological disorders (anemia, thrombocytopenia, eosinophilia, leukopenia, myelosuppression)**	**Gastrointestinal symptoms (diarrhea, vomiting, nausea, abdominal pain, dyspepsia)**	**Peripheral neuropathy**	**Electrolyte disturbance**	**Arthralgia**	**Psychiatric disorder**	**Dermatologi-cal** **symptoms**
Kwon et al.	17	N/R	N/R	N/R	N/R	N/R	1	N/R	N/R	N/R	N/R	N/R
Shi et al	85	59	21	13	10	24	15	16	5	3	9	2
Gao et al	39	35	9	2	6	15	11	8	11	2	6	N/R
Barvaliya et al.	11	6	N/R	5	4	N/R	33	4	N/R	9	4	18
Kashongwe et al.	3	1	N/R	2	5	14	15	15	N/R	N/R	N/R	15
Das et al.	1	N/R	N/R	N/R	N/R	N/R	N/R	N/R	N/R	N/R	N/R	N/R
Lee et al.	23	N/R	1	N/R	N/R	N/R	4	N/R	N/R	N/R	N/R	N/R
Kim et al.	7	28	N/R	N/R	N/R	N/R	32	N/R	N/R	34	N/R	8
Mase et al.	6	N/R	N/R	N/R	2	2	4	7	4	N/R	3	3
Olayanju et al.	12	36	N/R	8	59	43	30	30	N/R	20	9	N/R
Salhotra et al.	14	13	4	N/R	8	22	35	26	7	N/R	15	1
Kempker et al	1	1	N/R	N/R	N/R	N/R	N/R	N/R	N/R	N/R	N/R	N/R
Taune et al.	1	N/R	N/R	N/R	N/R	N/R	N/R	N/R	N/R	N/R	N/R	N/R
Ferlazzo et al	4	N/R	1	N/R	N/R	N/R	1	1	N/R	N/R	2	N/R
Hewison et al.	12	27	5	1	9	3	34	21	N/R	N/R	N/R	6
Ndjeka et al	10	N/R	N/R	N/R	N/R	N/R	N/R	N/R	N/R	N/R	N/R	N/R
Achar et al.	0	N/R	N/R	N/R	N/R	N/R	N/R	N/R	N/R	N/R	N/R	N/R
Guglielmetti et al	13	17	N/R	N/R	N/R	N/R	N/R	N/R	N/R	N/R	N/R	N/R
Borisov et al.	24/248	N/R	47/413	10/413	N/R	86/412	130/413	96/412	N/R	84/412	29/413	63/412
Conradie et al.	0	17	N/R	N/R	N/R	N/R	N/R	N/R	N/R	N/R	N/R	N/R
Tweed et al.	0	4	N/R	N/R	N/R	N/R	N/R	N/R	N/R	N/R	N/R	N/R
Moodlier et al.	N/R	3/30	N/R	N/R	N/R	8/30	3/30	N/R	N/R	6/30	N/R	4/30
Shim et al.	17	N/R	N/R	N/R	N/R	10	58	15	N/R	16	N/R	8
Pai et al.	109	N/R	N/R	175	1014	190	972	709	N/R	843	74	783
Nguyen et al.	4	13	N/R	1	N/R	3	3	3	5	5	N/R	5
Nguyen et al. (2)	44	27	4	6	3	10	6	1	18	1	2	1
Zhang et al.	3	9	12	N/R	2	2	N/R	3	2	N/R	N/R	N/R
Padmapriyadarsini et al.	40	77	N/R	N/R	N/R	85	N/R	69	N/R	N/R	N/R	97
Huerga et al.	7	13	40	12	16	31	5	134	9	N/R	N/R	N/R
Wu et al.	1	N/R	N/R	N/R	N/R	4	N/R	1	N/R	N/R	N/R	N/R
Li et al.	16	1	N/R	1	N/R	4	8	9	2	2	3	2
Hwang et al.	11	N/R	N/R	N/R	N/R	N/R	7	0	N/R	N/R	N/R	N/R
Combined effects	10.2 (6.3–17.1)	12.6 (7.8–19.6)	5.9 (3.8–8.9)	3.9 (2.9–5.1)	7.0(3.2–14.4)	12.5(7.4–20.5)	13.8(9.6–19.4)	13.9(10.0–18.8)	6.4(2.9–13.4)	10.1(7.0–14.3)	4.6(2.9–7.2)	9.8(5.8–16.0)
Heterogeneity	96 %	94 %	80 %	45 %	97 %	97 %	95 %	93 %	93 %	89 %	83 %	95 %
Beggs Test	0.12	0.06	0.05	0.30	0.84	0.76	0.43	0.23	0.75	0.05	0.64	0.09

**Table 5:** Summary of Subgroup Analysis.

**Subgroup**	**No. of Studies**	**No. of Patients**	**Treatment Success Rate(%)(95 % CI)**	**HeterogeneityI^2^ (%)**	**Begg’s Test Value of p**
Regimen Containing BDQ	33	9,241	78.4(74.2–82.1)	93	0.39
Regimen Containing BDQ + DLM	8	764	73.6(64.6–81.0)	73	0.27
**Types of Study**					
Observational Studies	36	9,934	76.9(72.9–80.4)	92	0.31
Experimental Studies	5	468	81.7(67.2–90.7)	86	0.80

BDQ = Bedaquiline.

DLM = Delaminid.

25 of the 36 observational studies (consisting of 7,088 patients) reported treatment failure rates. The pooled treatment failure rate was 4.7 % (95 % CI, 3.1––7.0); p = 0.00; I^2^ = 89 %. Additionally, 31 of the 36 observational studies (consisting of 8,046 patients) included in the review reported death rates. The pooled death rate was 8.9 % (95 % CI, 7.1–11.0); p = 0.00; I^2^ = 81 %. The Begg’s Test showed that there was no significant publication bias (p > 0.05; Table 5).

### 3.3 Outcomes in the experimental Studies

**Fig. 3:**
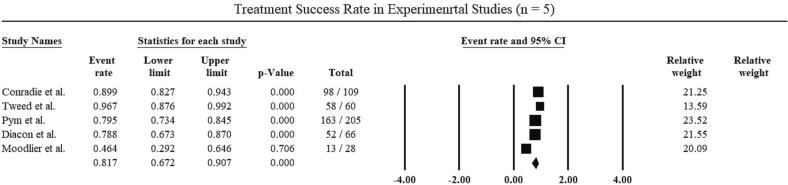
Treatment success rate of the pooled experimental studies.

The pooled results for the primary outcome i.e., the treatment success rate in 5 experimental studies included in the review was [81.7 % (95 % CI, 67.2–90.7); p = 0.00; I^2^ = 86 %; Fig. 3]. According to Begg’s Test, there was no evidence of significant publication bias (p > 0.05; Table 5).

2 of the 5 experimental studies (consisting of 139 patients) reported treatment failure rates. The pooled results of treatment failure rate was [3.6 % (95 % CI, 0.9–13.1); p = 0.00: I^2^ = 47 %]. 2 of the 5 experimental studies (consisting of 169 patients) reported death rates. The pooled death rate was [3.6 % (95 % CI, 0.6–19.6); p = 0.00; I^2^ = 51 %]. The Begg’s Test showed that there was no significant publication bias (p > 0.05; Table 5).

### 3.4 Adverse events (AEs)

Bedaquiline-containing regimens were associated with a spectrum of adverse events in patients being treated for drug-resistant tuberculosis. The corrected QT interval (QTc) was raised in 535 of the 7,562 patients [10.2 % (95 % CI, 6.3–17.1); p = 0.00: I^2^ = 92 %; Table 4]. But there is limited information on how many patients discontinued bedaquiline-based treatment as a result of this adverse event. Other AEs included liver disorders including hepatotoxicity (pooled rate, 12.6 %), renal disorders (pooled rate, 5.9 %), optic neuropathy including blurred vision (pooled rate, 3.9 %), ototoxicity including hearing loss (pooled rate, 7.0 %), hematological disorders (pooled rate, 12.5 %), gastrointestinal symptoms like nausea or vomiting (pooled rate, 13.8 %), peripheral neuropathy (pooled rate, 13.9 %), electrolyte disturbances (pooled rate, 6.4 %), arthralgia (pooled rate, 10.1 %), psychiatric disorders (pooled rate, 4.6 %), and dermatological disorders including acne (pooled rate, 9.8 %). The most common AEs are QTc prolongation, liver disorders, hematological disorders, gastrointestinal symptoms, arthralgia, dermatological disorders, and peripheral neuropathy [Table 4].

### 3.5 Subgroup Analysis

**Fig. 4:**
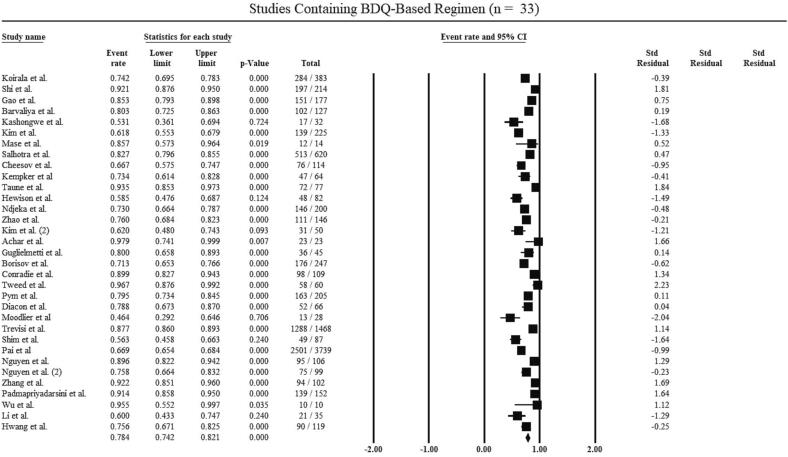
Treatment success rate for Bedaquiline-containing regimens.

**Fig. 5:**
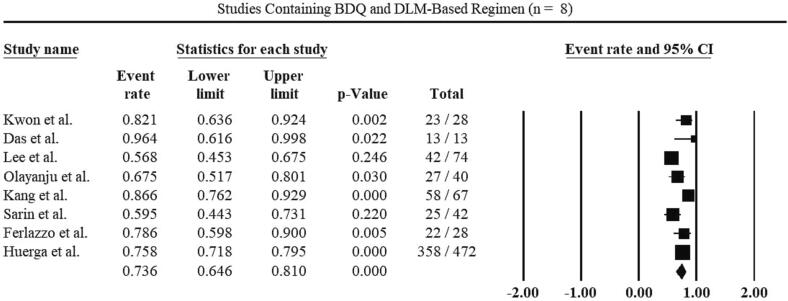
Treatment success rate for Bedaquiline and Delaminid-containing regimens.

The subgroup analysis of included studies in the review is shown in Table 5 based on treatment regimen and type of study. 33 of the 41 included studies with a total of 9,241 patients used bedaquiline-containing regimens and the pooled treatment success rate was 78.4 % (Fig. 4). 8 of the 41 studies with a total of 764 patients used treatment regimens including bedaquiline and delamanid. The pooled treatment success rate for their patients was 73.6 % (Fig. 5).

## 4 Discussion

To the best of our knowledge this *meta*-analysis is the largest and updated study conducted to date, involving a patient population of 10,402 individuals. The aim was to assess the safety, efficacy, and tolerability of treatment regimens containing bedaquiline in patients diagnosed with MDR-TB and XDR-TB who were undergoing treatment. This up-to-date *meta*-analysis provides evidence indicating that the use of regimens containing bedaquiline in 36 observational studies resulted in a treatment success rate of 76.9 %, the treatment failure rate was 4.7 % and death rate was 8.9 %. Pooled results of 5 experimental studies indicated that the success rate was 81.7 %, treatment failure rate and death rate was 3.6 %. The most common AEs are QTc prolongation, liver disorders, hematological disorders, gastrointestinal symptoms, arthralgia, dermatological disorders, and peripheral neuropathy. These findings suggest that bedaquiline-containing regimens are safe and effective for the treatment of MDR-TB and XDR-TB, with higher treatment success rates and lower mortality rates observed in experimental studies. However, large powered trials are warranted to confirm these findings.

The study found that treatment regimens containing bedaquiline had high treatment success rates and lower mortality rates. This can be attributed to the unique mechanism of action of this drug that inhibits the mycobacterial energy metabolism by specifically targeting ATP synthesis. It is effective against all forms of Mycobacterium tuberculosis, including active, dormant, replicating, non-replicating, intracellular, and extracellular bacteria. Amongst all anti-TB drugs approved, bedaquiline is the only drug, which targets the energy metabolism of mycobacteria [27]. Additionally, bedaquiline has a high volume of distribution, extensive tissue distribution, and high plasma protein binding (>99.9 %) and terminal half-life of ∼5.5 months [27]. The dynamic pharmacokinetics and unique mechanism of action make it a viable treatment option. Bedaquiline can be used in combination with other anti-TB drugs. This helps to improve the efficacy of the treatment regimen and reduce the risk of resistance [28]. However, further research is warranted to study drug interactions with bedaquiline.

The majority of the patients, specifically 9,321 out of a total of 10,402, underwent a treatment regimen lasting approximately six months. Among these patients, 6,690 individuals, accounting for approximately 72 % of the total, experienced successful outcomes from the treatment. The efficacy of BDQ-containing regimens for the treatment of BDQ has been substantiated by the significant rate of success observed over a 6-month treatment duration. Furthermore, the TB-PRACTECAL (Pragmatic Clinical Trial for a More Effective, Concise, and Less Toxic Multidrug-Resistant Tuberculosis Treatment Regimen[s]) [29] and STREAM-2 (The Evaluation of a Standard Treatment Regimen of Anti-Tuberculosis Drugs for Patients with Multidrug-Resistant Tuberculosis) [30] trials have provided evidence of the effectiveness of treatment regimens containing BDQ, with durations of 6–9 months. Collectively, these findings indicate that when combined with efficacious adjunctive medications, a duration of 6 months of BDQ may be satisfactory for a considerable number of patients [20].

The findings of our study indicate that bedaquiline-containing regimens exhibit efficacy and safety as a viable treatment choice for individuals afflicted with drug-resistant tuberculosis. The results of our study align with other individual trials and observational studies that have documented positive outcomes associated with the use of bedaquiline [10], [30], [31], [32], [33]. Our results are also consistent with the previous *meta*-analysis by Hatami et al. [21] in which the results of 29 studies recruiting almost 4000 patients were pooled and the average treatment success rate for observational and experimental studies was around 80 %. Another systematic review and *meta*-analysis conducted in 2021 found that bedaquiline exhibits the ability to attain a higher rate of culture conversion and a reduced risk of mortality in patients with drug-resistant tuberculosis [8].

Patients have demonstrated encouraging responses to bedaquiline, however, bedaquiline, like any medicine, can cause unwanted side effects that must be watched for and dealt with. Nausea, headache, arthralgia, and a loss of appetite were the most often reported adverse reactions to bedaquiline. Most of these were not serious enough to warrant stopping treatment with the medication [8]. One concerning thing is that bedaquiline can cause a condition called QT prolongation, which causes a delay in the heart's electrical activity and can cause irregular heart rhythms. When coupled with other medications that have the same effect or with other heart problems, QT prolongation can significantly raise the risk of sudden cardiac death. Therefore, patients with or at risk for QT prolongation should use bedaquiline with caution, and regular ECG monitoring is advised [34].

Additionally, bedaquiline can also cause hepatotoxicity manifested by increased liver enzymes, jaundice, or hepatitis. This can occur either independently of drug interactions or in conjunction with other hepatotoxic anti-tuberculosis medicat ions. Patients with or at risk for liver illness should use bedaquiline with caution, and routine liver function testing is advised [35]. However, bedaquiline's efficacy and safety in the treatment of multidrug-resistant tuberculosis (MDR-TB) were evaluated in an observational retrospective cohort research. The findings demonstrated that bedaquiline is a well-tolerated and safe medication with promising preliminary efficacy [36]. Since bedaquiline is a relatively new medication, studies assessing its long-term safety and efficacy are still in their infancy. Therefore, it is crucial to utilize bedaquiline in accordance with the standards and recommendations issued by the World Health Organization and other organizations. Bedaquiline is most effective when used in conjunction with other medications, under close medical supervision, and tailored to the specific needs of each patient [37]. Bedaquiline's cost-effectiveness and effect on drug-resistance patterns, as well as the appropriate dose, duration, and regimen, require more study.

Bedaquiline-containing regimens are effective in treating MDR-TB and XDR-TB, with higher treatment success rates. However, the treatment failure rates and death rates, although low, should not be ignored. One of the reasons could be inadequate adherence to treatment. Patients must take bedaquiline for the full course of treatment, which is 24 months [27]. If they do not take their medication as prescribed, the risk of treatment failure and death increases. Additionally, with the increased exposure of these medications to patients in all prescribed multidrug-resistant and rifampicin-resistant tuberculosis treatment regimens, resistance might increase in the population [38]. Moreover, bedaquiline can cause serious side effects, such as liver damage and QT prolongation [39]. These side effects can lead to treatment discontinuation, which increases the risk of treatment failure and death.

However, our study possesses certain limitations. Both experimental and observational studies were incorporated in this analysis, potentially resulting in heterogeneity. While the incorporation of observational studies in this *meta*-analysis adds vulnerability to residual bias, a seperate analysis was conducted for these study groups to assess their individual impact and implications. The main limitations include variations in the time period of drug usage and adjunctive dosing regimen of patients, as well as discrepancies in patient characteristics observed across different studies. Moreover, we exclusively considered published articles while excluding unpublished ones from our screening process. It is also important to note that the scope of this study was restricted to articles written exclusively in the English language, so excluding any publications in other languages from the review process.

In conclusion, this up-to-date *meta*-analysis of observational and experimental studies provides evidence that bedaquiline-containing regimens are safe and effective for the treatment of MDR-TB and XDR-TB. The treatment success rate was 76.9 % in observational studies and 81.7 % in experimental studies. The mortality rate was 8.9 % in observational studies and 3.6 % in experimental studies. The most common adverse events were QTc prolongation, liver disorders, hematological disorders, gastrointestinal symptoms, arthralgia, dermatological disorders, and peripheral neuropathy. However, large powered trials are warranted to confirm these findings.

## CRediT authorship contribution statement

**Obaid Ur Rehman:** Conceptualization, Data curation, Formal analysis, Methodology, Writing – original draft, Writing – review & editing. **Eeshal Fatima:** Data curation, Formal analysis, Writing – original draft, Writing – review & editing. **Abraish Ali:** Data curation, Formal analysis, Methodology, Writing – original draft, Writing – review & editing. **Umar Akram:** Data curation, Writing – original draft, Writing – review & editing. **Abdulqadir Nashwan:** . **Faryal Yunus:** Data curation, Writing – original draft, Writing – review & editing.

## Declaration of competing interest

The authors declare that they have no known competing financial interests or personal relationships that could have appeared to influence the work reported in this paper.

## References

[1] World Health Organization. [Internet]. *Tuberculosis (TB)*. [Assessed: August 20, 2023]. Available From: https://www.who.int/news-room/fact-sheets/detail/tuberculosis.

[2] World Health Organization. [Internet]. *2.3 drug-resistant TB*. [Assessed: August 20, 2023]. Available From:.https://www.who.int/teams/global-tuberculosis-programme/tb-reports/global-tuberculosis-report-2022/tb-disease-burden/2-3-drug-resistant-tb.

[3] Viney K., Linh N.N., Gegia M., Zignol M., Glaziou P., Ismail N. (2021). New definitions of pre-extensively and extensively drug-resistant tuberculosis update from the World Health Organization. Eur Respir J.

[4] World Health Organization. Global tuberculosis report 2013. World Health Organization; 2013.

[5] Conradie F., Diacon A.H., Ngubane N., Howell P., Everitt D., Crook A.M. (2020). Bedaquiline, pretomanid and linezolid for treatment of extensively drug resistant, intolerant or non-responsive multidrug resistant pulmonary tuberculosis. N Engl J Med.

[6] 4 Drug-resistant TB: treatment and treatment coverage (who.int).

[7] World Health Organization. WHO consolidated guidelines on drug-resistant tuberculosis treatment. World Health Organization; 2019. 30946559

[8] Wang M.G., Wu S.Q., He J.Q. (2021). Efficacy of bedaquiline in the treatment of drug-resistant tuberculosis: a systematic review and meta-analysis. BMC Infect Dis.

[9] Fox G.J., Menzies D. (2013). A review of the evidence for using bedaquiline (TMC207) to treat multi-drug resistant tuberculosis. Infect Dis Ther.

[10] Koirala S., Borisov S., Danila E., Mariandyshev A., Shrestha B., Lukhele N. (2021). Outcome of treatment of MDR-TB or drug-resistant patients treated with bedaquiline and delamanid Results from a large global cohort. Pulmonology.

[11] Kwon Y.S., Jeon D., Kang H., Yim J.J., Shim T.S. (2021). Concurrent use of bedaquiline and delamanid for the treatment of fluoroquinolone-resistant multidrug-resistant tuberculosis, a nationwide cohort study in South Korea. Eur Respir J.

[12] Shi L., Gao J., Gao M., Deng P., Chen S., He M. (2021). Interim Effectiveness and Safety Comparison of Bedaquiline-Containing Regimens for Treatment of Diabetic Versus Non-Diabetic MDR/XDR-TB Patients in China A Multicenter Retrospective Cohort Study. Infect Dis Ther.

[13] Gao M., Gao J., Xie L., Wu G., Chen W., Chen Y. (2021). Early outcome and safety of bedaquiline-containing regimens for treatment of MDR- and XDR-TB in China a multicentre study. Clin Microbiol Infect.

[14] Conradie F., Diacon A.H., Ngubane N., Howell P., Everitt D., Crook A.M. (2020). Treatment of Highly Drug-Resistant Pulmonary Tuberculosis. N Engl J Med.

[15] Tweed C.D., Dawson R., Burger D.A., Conradie A., Crook A.M., Mendel C.M. (2019). Bedaquiline, moxifloxacin, pretomanid, and pyrazinamide during the first 8 weeks of treatment of patients with drug-susceptible or drug-resistant pulmonary tuberculosis a multicentre, open-label, partially randomized, phase 2b trial. Lancet Respir Med.

[16] Pym A.S., Diacon A.H., Tang S.J., Conradie F., Danilovits M., Chuchottaworn C. (2016). Bedaquiline in the treatment of multidrug- and extensively drug-resistant tuberculosis. Eur Respir J.

[17] Diacon A.H., Pym A., Grobusch M.P., de los Rios J.M., Gotuzzo E., Vasilyeva I. (2014). Multidrug-resistant tuberculosis and culture conversion with bedaquiline. N Engl J Med.

[18] Shim T.S., Pai H., Mok J., Lee S.H., Kwon Y.S., Choi J.C. (2023). A prospective patient registry to monitor safety, effectiveness, and utilisation of bedaquiline in patients with multidrug-resistant tuberculosis in South Korea. BMC Infect Dis.

[19] Moodliar R., Aksenova V., Frias M.V.G., van de Logt J., Rossenu S., Birmingham E. (2021). Bedaquiline for multidrug-resistant TB in paediatric patients. Int J Tuberculosis Lung Disease.

[20] Trevisi L., Hernán M.A., Mitnick C.D., Khan U., Seung K.J., Rich M.L. (2023). Effectiveness of Bedaquiline Use Beyond Six Months in Patients with Multidrug-Resistant Tuberculosis. Am J Respir Crit Care Med.

[21] Hatami H., Sotgiu G., Bostanghadiri N., Abadi S.S., Mesgarpour B., Goudarzi H. (2022 May). Bedaquiline-containing regimens and multidrug-resistant tuberculosis: a systematic review and meta-analysis. J Bras Pneumol.

[22] Page MJ, McKenzie JE, Bossuyt PM, Boutron I, Hoffmann TC, Mulrow C D et al. The PRISMA 2020 statement: an updated guideline for reporting systematic reviews *BMJ* 2021; 372 :n71 doi:10.1136/bmj.n71. 10.1136/bmj.n71 PMC8005924 33782057

[23] World Health Organization (WHO) *Global tuberculosis report 2020.* Geneva: WHO; c2020. https://www.who.int/publications/i/item/9789240013131.

[24] Falzon D., Jaramillo E., Schünemann H.J., Arentz M., Bauer M., Bayona J. (2011). WHO guidelines for the programmatic management of drug resistant tuberculosis 2011 update. Eur Respir J.

[25] Begg C.B., Mazumdar M. (1994). Operating characteristics of a rank correlation test for publication bias. Biometrics.

[26] Joanna Briggs Institute . *Critical Appraisal Tools.* Adelaide, Australia: University of Adelaide; c2021. https://jbi.global/critical-appraisal-tools.

[27] Companion Handbook to the WHO Guidelines for the Programmatic Management of Drug-Resistant Tuberculosis. Geneva: World Health Organization; 2014. ANNEX 4, “How-to” guide on the use of bedaquiline for MDR-TB treatment. Available from: https://www.ncbi.nlm.nih.gov/books/NBK247434/. 25320836

[28] Shah N.S., Pratt R., Armstrong L., Robison V., Castro K.G., Cegielski J.P. (2008 Nov 12). Extensively drug-resistant tuberculosis in the United States, 1993–2007. JAMA.

[29] Nyang’wa B.T., Berry C., Kazounis E., Motta I., Parpieva N., Tigay Z. (2022). TB-PRACTECAL Study Collaborators. A 24-week all-oral regimen for rifampicin resistant tuberculosis. N Engl J Med.

[30] Goodall R.L., Meredith S.K., Nunn A.J., Bayissa A., Bhatnagar A.K., Bronson G. (2022). STREAM Study Collaborators. Evaluation of two short standardised regimens for the treatment of rifampicin-resistant tuberculosis (STREAM stage 2): an open-label, multicentre, randomised, non-inferiority trial. Lancet.

[31] Borisov S.E., Dheda K., Enwerem M., Leyet R.R., D'Ambrosio L., Centis R. (2017). Effectiveness and safety of bedaquiline-containing regimens in the treatment of MDR-and XDR-TB: a multicentre study. Eur Respir J.

[32] Guglielmetti L., Le Dû D., Jachym M., Henry B., Martin D., Caumes E. (2015). Compassionate use of bedaquiline for the treatment of multidrug-resistant and extensively drug-resistant tuberculosis: interim analysis of a French cohort. Clin Infect Dis.

[33] Conradie F., Bagdasaryan T.R., Borisov S., Howell P., Mikiashvili L., Ngubane N. (2022). Bedaquiline–pretomanid–linezolid regimens for drug-resistant tuberculosis. N Engl J Med.

[34] Hughes J.A., Solans B.P., Draper H.R., Schaaf H.S., Winckler J.L., Van der Laan L. (2022). Pharmacokinetics and safety of bedaquiline in human immunodeficiency virus (HIV)-positive and negative older children and adolescents with rifampicin-resistant tuberculosis. Clin Infect Dis.

[35] Bedaquiline side effects: common, severe, long term [Internet]. Drugs.com. Available from: https://www.drugs.com/sfx/bedaquiline-side-effects.html#refs.

[36] Taune M., Ustero P., Hiashiri S., Huang K., Aia P., Morris L. (2019). Successful implementation of bedaquiline for multidrug-resistant TB treatment in remote Papua New Guinea. Public Health Action.

[37] Medical. Bedaquiline [Internet]. Medical Dialogues. 2022. Available from: https://medicaldialogues.in/generics/bedaquiline-2722795.

[38] Mishra G.P., Mulani J. (2022). Implications of bedaquiline-resistant tuberculosis. Lancet Infect Dis.

[39] WebMD. (Interent). *Bedaquiline oral: Uses, side effects, interactions, pictures, warnings & dosing*. WebMD. Available From https://www.webmd.com/drugs/2/drug-163186/bedaquiline-oral/details#:∼:text=This%20medication%20may%20increase%20the,that%20may%20cause%20QT%20prolongation.

[40] Borisov S.E., Dheda K., Enwerem M., Romero Leyet R., D'Ambrosio L., Centis R. (2017). Effectiveness and safety of bedaquiline-containing regimens in the treatment of MDR- and XDR-TB a multicentre study. Eur Respir J.

[41] Barvaliya S.V., Desai M.K., Panchal J.R., Solanki R.N. (2020). Early treatment outcome of bedaquiline plus optimised background regimen in drug resistant tuberculosis patients. Indian J Tuberc.

[42] Kashongwe I.M., Mawete F., Mbulula L., Nsuela D.J., Losenga L., Anshambi N. (2020). Outcomes and adverse events of pre- and extensively drug-resistant tuberculosis patients in Kinshasa, Democratique Republic of the Congo A retrospective cohort study. PLoS One.

[43] Das M., Mamnoon F., Mansoor H., Meneguim A.C., Singh P., Shah I. (2020). New TB drugs for the treatment of children and adolescents with rifampicin-resistant TB in Mumbai, India. Int J Tuberc Lung Dis.

[44] Lee H.H., Jo K.W., Yim J.J., Jeon D., Kang H., Shim T.S. (2020). Interim treatment outcomes in multidrug-resistant tuberculosis patients treated sequentially with bedaquiline and delamanid. Int J Infect Dis.

[45] Kim J.H., Kwon O.J., Kim Y.S., Park M.S., Hwang S., Shim T.S. (2020). Bedaquiline in multidrug-resistant tuberculosis treatment Safety and efficacy in a Korean subpopulation. Respir Investig.

[46] Mase S., Chorba T., Parks S., Belanger A., Dworkin F., Seaworth B. (2020). Bedaquiline for the Treatment of Multidrug-resistant Tuberculosis in the United States. Clin Infect Dis.

[47] Olayanju O., Esmail A., Limberis J., Dheda K. (2020). A regimen containing bedaquiline and delamanid compared to bedaquiline in patients with drug-resistant tuberculosis. Eur Respir J.

[48] Salhotra V.S., Sachdeva K.S., Kshirsagar N., Parmar M., Ramachandran R., Padmapriyadarsini C. (2020). Effectiveness and safety of bedaquiline under conditional access program for treatment of drug-resistant tuberculosis in India An interim analysis. Indian J Tuberc.

[49] Chesov D., Heyckendorf J., Alexandru S., Donica A., Chesov E., Reimann M. (2021). Impact of bedaquiline on treatment outcomes of multidrug-resistant tuberculosis in a high-burden country. Eur Respir J.

[50] Kang H., Jo K.W., Jeon D., Yim J.J., Shim T.S. (2020). Interim treatment outcomes in multidrug-resistant tuberculosis using bedaquiline and/or delamanid in South Korea. Respir Med.

[51] Sarin R., Vohra V., Singla N., Singla R., Puri M.M., Munjal S.K. (2019). Early efficacy and safety of Bedaquiline and Delamanid given together in a “Salvage Regimen” for treatment of drug-resistant tuberculosis. Indian J Tuberc.

[52] Kempker R.R., Mikiashvili L., Zhao Y., Benkeser D., Barbakadze K., Bablishvili N. (2020). Clinical Outcomes Among Patients With Drug-resistant Tuberculosis Receiving Bedaquiline- or Delamanid-Containing Regimens. Clin Infect Dis.

[53] Taune M., Ustero P., Hiashiri S., Huang K., Aia P., Morris L. (2019). Successful implementation of bedaquiline for multidrug-resistant TB treatment in remote Papua New Guinea. Public Health Action.

[54] Ferlazzo G., Mohr E., Laxmeshwar C., Hewison C., Hughes J., Jonckheere S. (2018). Early safety and efficacy of the combination of bedaquiline and delamanid for the treatment of patients with drug-resistant tuberculosis in Armenia, India, and South Africa a retrospective cohort study. Lancet Infect Dis.

[55] Hewison C., Bastard M., Khachatryan N., Kotrikadze T., Hayrapetyan A., Avaliani Z. (2018). Is 6 months of bedaquiline enough Results from the compassionate use of bedaquiline in Armenia and Georgia. Int J Tuberc Lung Dis.

[56] Ndjeka N., Schnippel K., Master I., Meintjes G., Maartens G., Romero R. (2018). High treatment success rate for multidrug-resistant and extensively drug-resistant tuberculosis using a bedaquiline-containing treatment regimen. Eur Respir J.

[57] Zhao Y., Fox T., Manning K., Stewart A., Tiffin N., Khomo N. (2019). Improved Treatment Outcomes With Bedaquiline When Substituted for Second-line Injectable Agents in Multidrug-resistant Tuberculosis A Retrospective Cohort Study. Clin Infect Dis.

[58] Kim C.T., Kim T.O., Shin H.J., Ko Y.C., Hun Choe Y., Kim H.R. (2018). Bedaquiline and delamanid for the treatment of multidrug-resistant tuberculosis a multicentre cohort study in Korea. Eur Respir J.

[59] Achar J., Hewison C., Cavalheiro A.P., Skrahina A., Cajazeiro J., Nargiza P. (2017). Off-Label Use of Bedaquiline in Children and Adolescents with Multidrug-Resistant Tuberculosis. Emerg Infect Dis.

[60] Guglielmetti L., Jaspard M., Le Dû D., Lachâtre M., Marigot-Outtandy D., Bernard C. (2017). Long-term outcome and safety of prolonged bedaquiline treatment for multidrug-resistant tuberculosis. Eur Respir J.

[61] Pai H., Ndjeka N., Mbuagbaw L., Kaniga K., Birmingham E., Mao G. (2022). Bedaquiline safety, efficacy, utilization and emergence of resistance following treatment of multidrug-resistant tuberculosis patients in South Africa: a retrospective cohort analysis. BMC Infect Dis.

[62] Nguyen T.M.P., Le T.H.M., Merle C.S.C., Pedrazzoli D., Nguyen N.L., Decroo T. (2023). Effectiveness and safety of bedaquiline-based, modified all-oral 9–11-month treatment regimen for rifampicin-resistant tuberculosis in Vietnam. Int J Infectious Diseases: IJID.

[63] Nguyen T.M.P., Nguyen B.H., Hoang T.T.T., Nguyen H.A., Vu D.H., Nguyen M.H. (2022). Safety and effectiveness of all-oral and injectable-containing, bedaquiline-based long treatment regimen for pre-XDR tuberculosis in Vietnam. Front Pharmacol.

[64] Zhang S.J., Yang Y., Sun W.W., Zhang Z.S., Xiao H.P., Li Y.P. (2022). Effectiveness and safety of bedaquiline-containing regimens for treatment on patients with refractory RR/MDR/XDR-tuberculosis: a retrospective cohort study in East China. BMC Infect Dis.

[65] Padmapriyadarsini C., Vohra V., Bhatnagar A., Solanki R., Sridhar R., Anande L., Muthuvijaylakshmi M., Bhatia M., Jeyadeepa B., Taneja G., Balaji S., Shah P., Saravanan N., Chauhan V., Kumar H., Ponnuraja C., Livchits V., Bahl M., Alavadi U., Sachdeva K.S., for BEAT India Team (2022). Bedaquiline, Delamanid, Linezolid and Clofazimine for Treatment of Pre-extensively Drug-Resistant Tuberculosis. Clinical Infectious Diseases.

[66] Huerga H., Khan U., Bastard M., Mitnick C.D., Lachenal N., Khan P.Y. (2022). Safety and Effectiveness Outcomes From a 14-Country Cohort of Patients With Multi-Drug Resistant Tuberculosis Treated Concomitantly With Bedaquiline, Delamanid, and Other Second-Line Drugs. Clin Infect Dis.

[67] Wu H.Y., Tian Y., Wang X.D., Sun J.S., Fan L.C., Chen M.X. (2022). Twenty-four-week interim outcomes of bedaquiline-containing regimens in treatment of adolescents with rifampicin-resistant tuberculosis: A retrospective cohort study in China. J Paediatr Child Health.

[68] Li J., Yang G., Cai Q., Wang Y., Xu Y., Zhang R. (2021). Safety, efficacy, and serum concentration monitoring of bedaquiline in Chinese patients with multidrug-resistant tuberculosis. Int J Infectious Diseases: IJID.

[69] Hwang H., Kang H., Kwon Y.S., Jeon D., Shim T.S., Yim J.J. (2021). Outcomes of Multidrug-Resistant Tuberculosis Treated With Bedaquiline or Delamanid. Clin Infect Dis.

