# Supplementary material for: Efficacy and safety of bedaquiline containing regimens in patients of drug-resistant tuberculosis: An updated systematic review and meta-analysis
Source: J Clin Tuberc Other Mycobact Dis. 2023 Dec 1;34:100405. doi: 10.1016/j.jctube.2023.100405 (PMC10750101; doi:10.1016/j.jctube.2023.100405)
Supplement: Supplementary data 1 [file mmc1.docx]

**Supplemental Table S1**: Detailed Search Strategy

| **Database** | **Search String** |
| --- | --- |
| PubMed MEDLINE  293 Results | (("bedaquiline"[Supplementary Concept] OR "bedaquiline"[All Fields]) AND ("MDR-TB"[All Fields] OR ("tuberculosi"[All Fields] OR "tuberculosis"[MeSH Terms] OR "tuberculosis"[All Fields] OR "tuberculoses"[All Fields] OR "tuberculosis s"[All Fields])) AND ("efficacies"[All Fields] OR "efficacious"[All Fields] OR "efficaciously"[All Fields] OR "efficaciousness"[All Fields] OR "efficacy"[All Fields] OR ("effect"[All Fields] OR "effecting"[All Fields] OR "effective"[All Fields] OR "effectively"[All Fields] OR "effectiveness"[All Fields] OR "effectivenesses"[All Fields] OR "effectives"[All Fields] OR "effectivities"[All Fields] OR "effectivity"[All Fields] OR "effects"[All Fields]))) AND (2021:2023[pdat]) |
| Scopus  40 Results | [bedaquiline AND ( mdr-tb OR tuberculosis ) AND ( efficacy OR effectiveness ) AND ( clinical AND trials ) AND culture AND conversion] |
| Cochranelibrary.com  400 Results | Bedaquiline AND Tuberculosis AND Efficacy OR Effectiveness AND Culture Conversion AND Trials |
